# Patellar resurfacing as a prognostic variable in total knee arthroplasty: a two‑decade retrospective cohort (2000‑2020)

**DOI:** 10.1186/s12891-025-09076-y

**Published:** 2026-01-13

**Authors:** Diego Laverde Osorio, Luis David Marcial Barba, Nelva Garduza Leyva, Efrain Diaz Borjon, Juan Montejo Vargas, Georges Jirjis Makdissy Salomon, Christian Hazel Hernandez Romero

**Affiliations:** 1 Instituto Nacional Ciencias Medicas Nutrición Salvador Zubiran Hip and Knee Joint Reconstruction service Contribution/s: Data Curation, Universidad Nacional Autonoma de Mexico, Vasco de Quiroga 15, Belisario Domínguez Secc 16 Tlalpan, Ciudad de México, 14080 México; 2 Instituto Nacional Ciencias Medicas Nutrición Salvador Zubiran Head of Hip and Knee Joint Reconstruction service Contribution/s: Supervision, Universidad Nacional Autonoma de Mexico, Vasco de Quiroga 15, Belisario Domínguez Secc 16, Tlalpan, Ciudad de México CDMX, 14080 México; 3 Instituto Nacional Ciencias Medicas Nutrición Salvador Zubiran Hip and Knee Joint Reconstruction service Contribution/s: Conceptualization, Universidad Nacional Autonoma de Mexico, Vasco de Quiroga 15, Belisario Domínguez Secc 16, Tlalpan, Ciudad de México CDMX, 14080 México; 4 Instituto Nacional Ciencias Medicas Nutrición Salvador Zubiran Hip and Knee Joint Reconstruction service Contribution/s: Writing - Review & Editing, Universidad Nacional Autonoma de Mexico, Vasco de Quiroga 15, Belisario Domínguez Secc 16, Tlalpan, Ciudad de México, CDMX, 14080 México; 5 Instituto Nacional Ciencias Medicas Nutrición Salvador Zubiran Hip and Knee Joint Reconstruction service Contribution/s: Writing - Original Draft, Universidad Nacional Autonoma de Mexico, Vasco de Quiroga 15, Belisario Domínguez Secc 16, Tlalpan, Ciudad de México, CDMX, 14080 México

**Keywords:** Total knee arthroplasty, Patellar resurfacing, Anterior knee pain, Aseptic loosening, Absolute risk reduction, Number needed to treat

## Abstract

**Background:**

The impact of patellar resurfacing (PR) on long-term outcomes following primary total knee arthroplasty (TKA) remains a topic of debate.

**Methods:**

This study examined a retrospective cohort of 334 primary total knee arthroplasties (TKAs) performed between 2000 and 2020 at a specialized hospital. The surgeries were conducted from January 2000 to December 2020, allowing for a maximum potential follow-up period of 20 years. The primary endpoint assessed was the rate of any-cause revision. Secondary endpoints included anterior knee pain, aseptic loosening, patient-reported outcomes (Western Ontario and McMaster Universities Osteoarthritis index and Oxford Knee Score), and complications. Kaplan-Meier estimates and multivariable Cox models, adjusted for age, body mass index, and inflammatory arthropathy, were utilized.

**Results:**

PR yielded an absolute risk reduction (ARR) of 83% for anterior knee pain (2.2% in the PR group vs. 85.2% in the WPR group; number needed to treat ≈ 1–2) and an ARR of 7.5% for aseptic loosening (4.4% vs. 11.9%; NNT = 14). The overall revision rates were 5.1% for PR and 6.7% for WPR, demonstrating no significant differences (hazard ratio 0.65, 95% confidence interval 0.30–1.42).

**Conclusions:**

Patellar resurfacing significantly alleviates anterior knee pain and decreases the risk of aseptic loosening without raising the overall revision rate. These findings advocate for a selective resurfacing approach targeting patellae at a higher risk of pain rather than adopting a routine or universal resurfacing strategy.

**Trial registration:**

Not applicable, this study is an observational retrospective cohort; no prospective registration was required.

**Supplementary Information:**

The online version contains supplementary material available at 10.1186/s12891-025-09076-y.

## Background

Whether to resurface the patella during total knee arthroplasty (TKA) remains a topic of controversy, as clinical and functional studies have not consistently shown a clear advantage for either approach. Outcome reporting has diversified, yet no single metric or biomarker dictates the decision. In a registry review of > 325,000 total knee arthroplasties (TKAs), Lan et al. found that Knee Society (KSS) and Western Ontario and McMaster Universities Osteoarthritis index (WOMAC) scores predominate. However, surgeons still employ a dozen different patient-reported outcome measures (PROMs), and fewer than half of the cases capture patient completed instruments [1]. Compounding the problem, a knee with osteoarthritis (OA) is now recognized as a heterogeneous, whole-organ disease influenced by biomechanics, inflammation, and genetics [2]; radiographic grade, cartilage quality, and symptom burden seldom align, making a universal resurfacing rule unrealistic. Therefore, the decision should be individualized based on the patient’s specific factors.

Evidence syntheses mirror this uncertainty. Schindler’s 2012 review of early randomized trials reported highly variable functional outcomes and complication rates [3]. The most significant contemporary metaanalysis by Tang et al. detected only modest KSS gains (≈ 0.6–1.4 points) and wide heterogeneity, across other PROMs [4]. At the same time, Fleaca et al. found no functional scale that consistently distinguished techniques [6]. A broader review by Grela et al. rated functional benefits of resurfacing as minimal and lowcertainty, although both Tang and Grela confirmed reductions in anterior knee pain and patellarelated reoperation [4] [7]. Those clinical advantages underpin the economic model of Parsons et al., who calculated a 10-year saving of approximately £104 per case and a non-significant gain of 0.19 quality-adjusted life years (QALYs) when patella is resurfaced [10]. Findings from cruciateretaining (CR) cohorts further illustrate the nuance. In 43 bilateral TKAs, Ko et al. resurfaced only patellae with Outerbridge ≥ 2 damage and found no patient-reported outcome measure (PROM) differences at 5.7 years [15]. A matched series of 500 CR-TKAs by Noh et al. reported that anteriorkneepain scores decreased significantly after TKA in both groups, with no between group difference [16]. Complication profiles differ resurfacing adds the risk of fracture or maltracking, whereas retention can lead to progressive patellofemoral arthritis and necessitate secondary resurfacing. Norwegian registry data indicate that secondary resurfacing relieves pain in approximately two-thirds of patients, but it confers a lower ten-year survival rate than primary resurfacing [8].

We will use the standard epidemiological measures of odds ratio (OR), relative risk (RR), and hazard ratio (HR) to interpret the literature and our data. These measures are used to compare absolute risk, relative risk, and risk over time. We quantified the strength of the relationship between patellar resurfacing and outcomes using the odds ratio (OR), risk ratio (RR), and hazard ratio (HR). The OR compares the probability of an event occurring in different groups, the RR compares cumulative incidence, and the HR, estimated using Cox regression, compares instantaneous rates over time. All estimators are presented with their 95% confidence intervals. Values greater than 1 indicate a higher risk in the exposed group, while values less than 1 indicate a lower risk.

We hypothesize that, within a 20-year single-center cohort of primary TKA, knees treated with PR will experience [1] a lower overall rate of postoperative complications and [2] a significantly reduced risk of aseptic loosening and subsequent revision, compared with non-resurfaced knees, independent of implant design, surgical era, and patient-related factors. To date no longitudinal, single-centre study has been specifically designed to test patellar resurfacing as an independent prognostic factor for overall aseptic loosening after primary TKA. Our investigation therefore addresses a persistent evidence gap by applying multivariable survival modelling to a uniform surgical cohort with up to two decades of follow-up.

## Methods

This retrospective, comparative cohort study was conducted at the Hip‑and‑Knee Joint Reconstruction Service of a tertiary referral hospital in Mexico City. All primary total knee arthroplasties (TKA) performed between 1 January 2000 and 31 December 2020 were eligible. The operations were carried out by three hip‑and‑knee arthroplasty surgeons who followed an unchanged surgical protocol throughout the two‑decade study period. Surgeons followed a pragmatic algorithm: resurfacing was selected when patellar cartilage was Outerbridge ≥ 2, tracking remained centered after trial reduction, or the patient had a history of disabling anterior knee pain. Patients were included if they were ≥ 18 years old, had Kellgren–Lawrence grade IV gonarthrosis and underwent their index TKA in our unit. Those who died during follow‑up, were first operated elsewhere or declined informed consent were excluded. Of 334 eligible individuals, 23 died of unrelated causes, 30 had been treated in another center and 10 declined participations, leaving 271 patients for analysis; the median follow‑up was 8 years (inter‑quartile range 4–15). The exposure of interest was patellar management, classified as resurfacing (PR) or non‑resurfacing (WPR) according to the operative note. Among the patients who underwent bilateral TKA, both knees were treated with the same patellar strategy (received bilateral PR and bilateral WPR); no patient intentionally received a mixed approach. Additionally, preoperative pelvic radiographs were reviewed to document hip osteoarthritis (Kellgren-Lawrence classification). Cases with a grade of at least III were analyzed in a sensitivity model.

The primary outcome was any‑cause revision. Secondary outcomes comprised anterior knee pain, aseptic loosening, periprosthetic joint infection, arthrofibrosis, instability, fracture and patient‑reported scores (WOMAC, Oxford Knee Score) together with pain intensity on a 0–10 Numeric Rating Scale. Age, sex, body‑mass index, laterality and rheumatic comorbidities (rheumatoid arthritis, systemic lupus erythematosus, ankylosing spondylitis) were recorded as potential confounders. Data were abstracted independently by two reviewers using a piloted form; disagreements were resolved by consensus. Continuous variables are reported as mean ± standard deviation or median (inter‑quartile range) and categorical variables as number and percentage. Group comparisons used the t test or Mann–Whitney U test for continuous data and the χ² or Fisher exact test for categorical data. Associations between patellar status and outcomes were expressed as odds ratios (OR) and risk ratios (RR), with a 95% of confidence interval (IC). Time‑to‑event data were analyzed with Kaplan–Meier curves compared by the log‑rank test; multivariable Cox regression, adjusted for anterior knee pain and aseptic loosening, provided hazard ratios (HR). Proportionality of hazards was verified with Schoenfeld residuals. Absolute risk reduction (ARR) was calculated as the difference in event incidence between WPR and PR groups, and the number needed to treat (NNT) as the inverse of the ARR. Confidence intervals at the 95% level for the ARR were obtained with the Newcombe–Wilson method; when this interval encompassed 0 the NNT was reported as not significant (NS). All analyses were performed with Stata 15 (Stata Corp, College Station, TX, USA) using two‑sided tests and a significance threshold of 0.05. No key parameter had more than 5% missing values.

We implemented several measures to minimize bias. Selection bias was reduced by including all consecutive primary TKAs performed between January 2000 and December 2020, with no exclusions based on outcome. Information bias was minimized through double data entry and cross-checking with the institutional arthroplasty registry; a 10% random audit revealed a 0.8% discordance rate. Outcome misclassification was minimised by having two fellowship-trained arthroplasty surgeons independently adjudicate revision causes, blinded to resurfacing status; disagreements were resolved by consensus. Attrition bias was addressed using Kaplan–Meier survival methods with right-censoring at the last documented follow-up or death. Finally, confounding was controlled a priori through multivariable Cox models adjusting for age, sex, BMI, diagnosis, implant model, and calendar period. This investigation was designed as a retrospective cohort study that included all consecutive primary TKAs performed between January 1, 2000, and December 31, 2020. This approach yielded a pragmatic sample of 239 knees. A priori sample size calculations were not feasible. A post-hoc assessment using Schoenfeld’s method indicated that the 22 documented cases of aseptic loosening provide approximately 60% power (α = 0.05, two-sided) to detect the observed hazard ratio (HR = 0.65). While this sample may be underpowered for detecting smaller effect sizes, the number of events is comparable to or greater than those found in previous single-center cohorts, ensuring that all available data was analyzed without loss of selection. This study was conducted with the utmost integrity and respect for ethical standards and was approved by our department’s Research and Ethics Committee. It did not receive specific funding from the public, commercial, or nonprofit sectors.

## Results

**Fig. 1:**
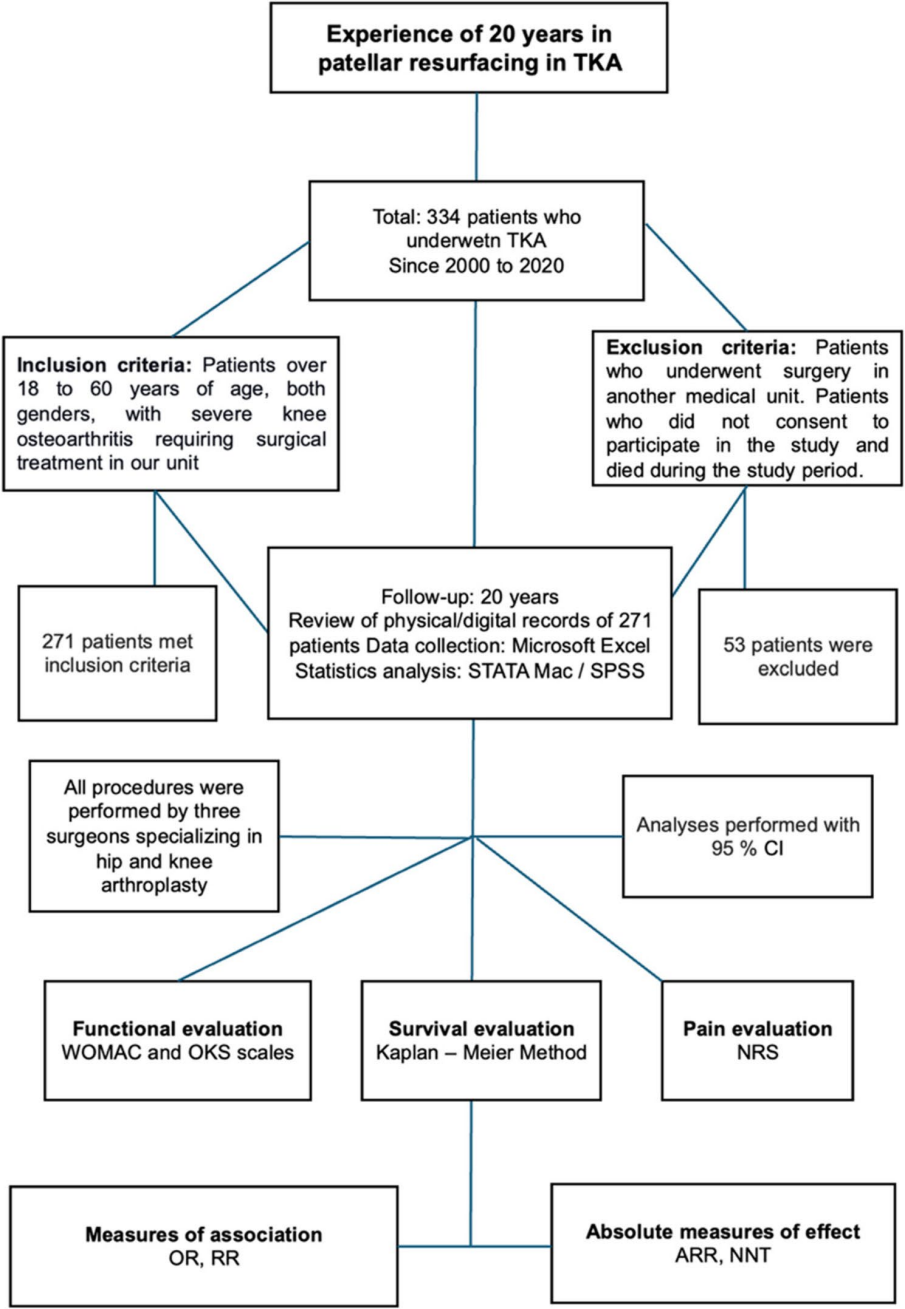
The methodology for patients who underwent PR techniques in TKA over 20 years

According to our study a total of 334 patients were documented over the past 20 years in Latin American population. Of those, 271 met the inclusion criteria. Twenty-three patients died from causes unrelated to TKA, 30 patients underwent surgery for TKA at another medical unit, and 10 patients decided not to participate in the study for personal reasons. The method used to select patients eligible for this study is shown in Fig. 1.

Methodology used for all participants related to the PR and WPR technique in TKA. Two hundred seventy-one patients who underwent TKA met the inclusion criteria, and 66 were excluded.

Age was analyzed as a continuous variable (mean ± SD) and, for descriptive purposes, was stratified into five ten-year bands that correspond to Table 1. Body mass index (BMI) was assessed both continuously and according to standard WHO categories: < 25 kg·m⁻² (normal), 25–29.9 kg·m⁻² (overweight), 30–34.9 kg·m⁻² (obesity class I), and ≥ 35 kg·m⁻² (obesity class II/III). Table 1 shows the distribution by gender and age ranges; we reported that degenerative joint pathology becomes more prevalent with age, particularly after age 40. On the other hand, regarding the laterality of TKA, 59.4% underwent surgery in one extremity, and 40.6% underwent TKA in both extremities at different surgical events, not during the same surgical event.

**Table 1:** Distribution of age groups of patients who underwent TKA

Age groups	Gender		Total (%)
	Female (%)	Male (%)	
20–29 years	2	0	1
30–39 years	5	2	4
40–49 years	14	13	14
50–59 years	20	24	21
> 60 years	59	62	59

The frequency among the total number of cases was 271 patients

The distribution frequency percentage is presented by age group and gender

We documented the technical characteristics included in the post-surgical note and the Hip and Knee Joint Reconstruction Service database from the medical record. According to this database, we categorized patients who underwent TKA into two study groups for the development of this project. Our population included 271 patients, 216 of whom were women (80%) and 55 of whom were men (20%). The two study groups related to TKA, were the PR group and the WPR group. Both groups had a higher proportion of female patients: 79.4% in the PR group and 80% in the WPR group. The most prevalent comorbidity in both groups was rheumatoid arthritis (RA): 81% of patients in the PR group and 89% of patients in the WPR group had RA. Similarly, systemic lupus erythematosus (SLE) occurred more frequently in the PR group (18%) than in the WPR group (10%). Table 2 compares comorbidities between the two study groups. No significant differences were observed regarding age and body mass index. However, joint wear and tear and symptomatology increase with age, leading to joint replacement.

**Table 2:** Comorbidities of PR and WPR groups after TKA

Comorbidities	PR group	WPR group	*P*-value *
Number of patients	136	135	
Rheumatoid arthritis	93	102	0.190
Systemic lupus erythematosus	24	13	0.360
Ankylosing spondylitis	2	2	-

*P-values were obtained with the χ^2^ test or Fisher’s exact test

Table 3 shows that the WPR group reported persistent anterior knee pain, aseptic loosening, and arthrofibrosis. Additionally, a higher frequency of revision surgeries was observed in the WPR group; however, no significant differences were noted. The most frequent complications reported in the latter group were periprosthetic infection and aseptic loosening. These results suggest that PR reduces the risk of anterior knee pain and septic loosening compared to the levels reported by the WPR group.

**Table 3:** Post‑operative complications in knees with PR versus WPR in TKA

Complications	PR group	WPR group	*P*-value*
Aseptic loosening	4	12	0.01
Periprosthetic joint infection	5	5	0.91
Arthrofibrosis	4	8	0.24
Posterior instability	0	1	0.30
Medial instability	0	2	0.14
Patellar tendinitis	0	1	0.30
Periprosthetic fracture	2	2	0.66
Peroneal nerve injury	2	2	0.95
Anterior knee pain	2	85	< 0.001
Revision surgery	7	9	0.54

Data are number of knees in each cohort; percentages are expressed relative to the total knees in that group (PR = 136, WPR = 135). *P values were obtained with a two-sided z-test for the difference in proportions; values < 0.05 were considered statistically significant

To provide context for aseptic loosening, we analyzed the distribution of prosthetic systems utilized during the study period and their relationship with the resurfacing techniques (PR vs. WPR). The majority of procedures employed Vanguard, AGC, or Scorpio models. Implants with three or fewer cases were categorized as “Other/Not consigned.” Table 4 presents this distribution along with the frequency of aseptic loosening recorded for each group.

**Table 4:** Implant and incidence of aseptic loosening in knees with RP versus WPR in TKA

Implants employed	Surgeries in PR group	Aseptic loosening PR %	Surgeries in WPR group	Aseptic loosening WPR %
Vanguard Biomet	60	0/0%	59	0/0%
AGC	32	2/6.3%	40	1/2.5%
Scorpio	17	0/0%	19	0/0%
Johnson & Johnson	1	0/0%	3	0/0%
PFC	2	0/0%	2	0/0%
Attune	2	0/0%	0	0/0%
Axims	0	0/0%	1	0/0%
Anthem Smith&Nephew	0	0/0%	1	0/0%
Other/not reported	22	0/0%	9	2/22.2%

Distribution of prosthetic systems and episodes of aseptic loosening in arthroplasties with PR and WPR. The Other/not reported category groups cases in which the implant could not be identified due to incomplete operative records

To examine changes in surgical practices over time, we organized the 271 TKA procedures into four chronological periods: 2000–2005, 2006–2010, 2011–2015, and 2016–2020. For each period, we calculated both the number and proportion of cases that involved PR compared to those that did not (WPR). In Table 5, we reported the use of PR remained fairly stable across these eras, fluctuating between 46.2% and 53.6%. Although minor variations were noted, no definitive upward or downward trend was observed. This indicates that decisions regarding resurfacing were likely influenced by the surgeon’s discretion or specific case factors, rather than changes in institutional protocols or implant types.

**Table 5:** Five‑year era trends in the use of PR during primary TKA

Years of study (Eras)	Total TKAs	PR cases (%)	WPR cases (%)
2000–2005	55	28 (50.9^a^)	27 (49.1^a^)
2006–2010	45	21 (46.7^a^)	24 (53.3^a^)
2011–2015	69	37 (53.6^a^)	32 (46.4^a^)
2016–2020	93	43 (46.2^a^)	50 (53.8^a^)

This table summarizes the number and proportion of TKAs across four chronological 5-year eras. The proportion of PR remained relatively stable throughout the 20-year study period, without evidence of a clear increasing or decreasing trend

^a^Frequency of presentation, reported in percentage

Over a follow-up period of more than 20 years, the use of PR reduced the incidence of aseptic loosening by 7.4% (95% CI 1.0–13.9), resulting in a number needed to treat (NNT) of 14 knees (95% CI 7–101). Additionally, PR significantly decreased the absolute risk of anterior knee pain by 83% (95% CI 76.5–89.5), which corresponds to an NNT of 2. In other words, treating two knees with PR prevents one case of anterior knee pain, while treating 14 knees prevents one episode of aseptic loosening.

Pain severity was measured using an NRS and categorized as mild (0–3), moderate (4-6), and severe (7-10). The WOMAC index score (ranging from 0 to 96) was grouped into quartiles based on our sample distribution: Q1 ≤ 34, Q2 35–51, Q3 52–68, and Q4 ≥ 69. The OKS was evaluated using its validated clinical cut-points: poor (< 20), moderate (20-29), good (30–39), and excellent (40–48). Based on our results, the PR group had better functional outcomes. Table 6 shows improvement in both study groups following TKA. For the OKS scale, the evaluation parameters are as follows: a score of 40 to 48 points indicates an excellent outcome because the patient has few limitations when performing daily activities; a score of 30 to 39 points indicates a good outcome because the patient may have limitations; a score of 20 to 29 points indicates a moderate outcome due to pain and functional limitations of varying degrees; and a score of less than 20 points indicates a poor outcome because pain is intense and knee function is severely affected.

**Table 6:** Preoperative and postoperative ranges of functional and pain scores in knees with PR versus WPR

Evaluation scale	Study phase	PR group	WPR group	*P*-value*
		Range of scores per scale		
WOMAC	Preoperative	22–94	22–89	0.010
	Postoperative	0–70	0–70	< 0.001
OKS	Preoperative	2–34	2–29	0.860
	Postoperative	14–48	10–48	< 0.001
NRS	Preoperative	4–89	3–10	0.690
	Postoperative	0–6	0–6	< 0.001

This is a report of the range of scores of the WOMAC and OKS functional scales, as well as a pain assessment

*The P value was estimated using the student's t-test

**Fig. 2:**
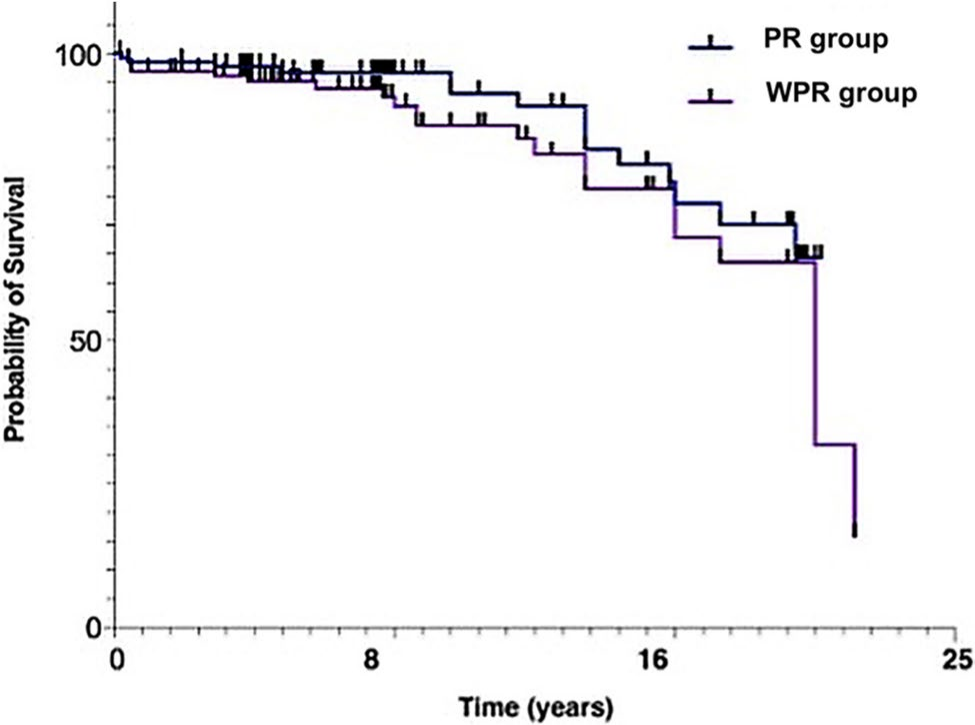
Kaplan–meier implantsurvival curves for knees with PR versus WPR. The graph shows revision-free survival of 136 PR knees (blue) and 135 WPR knees (purple) over a maximum follow-up of 24 years; vertical ticks denote censored observations. At 8 years the probability of survival was 96.7 % for PR versus 90.7 % for WPR; at 16 years it was 73.8 % versus 67.8 %

Both groups showed postoperative improvement; however, patients undergoing PR techniques significantly improved functional assessment and decreased postoperative pain assessment. Figure 2 shows that the probability of survival at 8.3 years is 96.7% for the PR group and 90.7% for the WPR group. After 16.6 years, the likelihood of survival decreases to 73.8% and 67.8% for the PR and WPR groups, respectively. No statistical significance was reported between the two groups.

A prognostic variable enables us to anticipate or predict a future clinical event or outcome, such as complications like loosening, anterior knee pain, periprosthetic infection, and prosthesis failure, in our study. In the cohort of 271 primary TKAs (136 with PR and 135 WPR, resurfacing was strongly associated with lower mechanical failure of the femorotibial construct and, even more strikingly, with the relief of patellofemoral symptoms. The incidence of aseptic loosening fell from 11.9% in WPR to 4.4% in PR, yielding a RR of 0.37 (95% CI 0.15–0.92) and an OR of 0.34 (0.13–0.91). Expressed clinically, this translates to an ARR of 7.5% (1.0–13.9) and a NNT of 14 (7–101): on average, fourteen resurfacings prevent one loosening event over the first decade after surgery. The benefit is even more pronounced for anterior knee pain. Only 3 of 136 PR knees reported persistent pain versus 115 of 135 WPR knees, giving an RR of 0.03 (0.01–0.08) and an OR of 0.004 (0.001–0.014). The ARR is 83% **(**76.5–89.5), so virtually every patella that is resurfaced spares a patient from disabling anterior pain (NNT ≈ 1–2). This large effect size explains the significantly greater gains observed in PROMs (Δ WOMAC − 44 ± 18 vs. −29 ± 19; Δ OKS + 27 ± 9 vs. + 19 ± 12; both *p* < 0.001) and supports the biomechanical rationale of improved patellofemoral congruence. By contrast, allcause revision was uncommon (7/136 PR vs. 9/135 WPR); the RR of 0.77 (0.30–2.01) and OR of 0.76(0.27–2.10) did not reach statistical significance, and the95% CI for the ARR of 0.6% (−4.1–7.1) crossed zero (NNT = 63, nonsignificant). Thus, while PR does not measurably affect overall revision rates within the followup available, it does confer a clinically relevant and statistically robust reduction in the two outcomes that most directly influence patient satisfaction—patellofemoral pain and aseptic loosening—without introducing additional risk. These data justify a selective resurfacing strategy that prioritizes patellar replacement in patients at higher risk of patellofemoral pain or high mechanical demand, while acknowledging that larger series are required to clarify its effect on long-term revision. Table 7, reported the relation between the evaluation of the association measures and resurfacing techniques.

**Table 7:** Effect of patellar resurfacing on major postoperative outcomes

Main outcomes after TKA	PR *n*/*N* (%)	WPR *n*/*N* (%)	RR (95% CI)	ARR %	NNT
Aseptic loosening	6/136 (4.4)*	16/135 (11.9)*	0.37 (0.15–0.92)*	–7.5	14
Anterior knee pain	3/136 (2.2)*	115/135 (85.2)*	0.03 (0.01–0.08)*	–83.0	2
Revision surgery	7/136 (5.1)*	9/135 (6.7)*	0.77 (0.30–2.01)*	–1.6	NS

*Statistically significant difference between PR and WPR (two-sided χ² or Fisher’s exact test; α=0.05). RR < 1 and negative ARR indicate the benefit of patellar resurfacing. NNT is reported only when the difference is significant (NNT = 1/|ARR|); otherwise shown as not significant (NS)

We assessed the time to revision surgery using the Cox regression model. Table 6 presents the multivariate model where the absence of resurfacing was not significantly influenced by revision (HR 2.33; *p* = 0.76). Aseptic loosening emerged as the sole independent predictor (HR 16.7; *p* < 0.001). To account for clinical confounding factors, anterior pain, aseptic loosening, and periprosthetic infection were included as dichotomous covariates. These findings confirm the clinical magnitude previously discussed in the ARR and NNT sections; these findings are reported in Table 8.

**Table 8:** Cox regression model related to resurfacing in TKA

Variable	HR	IC 95%	*P*-value*
WPR VS PR	2.33	0.01–500	0.76
Anterior knee pain	1.15	0.005–250	0.96
Aseptic loosening	16.7	4.35–50.0	< 0.001
Periprosthetic joint infection	3.85	0.92–16.7	0.07

The PR group was used as the reference in the Cox regression analysis

*The P-value was calculated using the Wald test

No difference in periprosthetic joint infection was observed between the groups, suggesting that the additional patellar step does not prolong operative time enough to influence infection risk.

## Discussion

Our findings must be interpreted against an evidence base that, despite decades of work, still offers no uniform indication for PR. Early randomized work, summarized by Schindler et al. in 2012, already showed that functional scores and complication rates varied so widely that neither routine resurfacing nor systematic retention could be endorsed [3]. Subsequent evidence syntheses keep the debate alive. The largest trialsequential metaanalysis to date—Tang et al. in 2023, 50 randomized controlled trials (RCTs), found only modest gains of ≈ 0.6 points in the KSSclinical and ≈ 1.4 points in the KSSfunctional subscales, while other PROMs domains (OKS, WOMAC, KOOS) remained heterogeneous [4]. A study reported by Fleaca et al. in 2022, likewise failed to identify any functional scale that consistently separated techniques [6]. Even so, both Tang et al. in 2023 and the broader systematic review of Grela et al. 2022 [7] confirmed that resurfacing lowers anterior knee pain and patellarelated reoperations [4], [9]; benefits that underpin the cost effectiveness model, reported by Parsons et al. in 2021, who calculated a 10 year saving of ≈ £104 per case and an incremental 0.19 QALYs despite only minor PROM differences [10].

According to a 2019 study by Teel et al., the PR technique is associated with a reduced need for revision and improved postoperative functional scores in primary TKA [9].

Modern implant series reinforce these clinical gains. Benazzo et al., in 2020, observed higher composite functional ratings and fewer patellofemoral complaints with contemporary component design and precise alignment [5]. When resurfacing is deferred, a rescue effect is still possible: a casebased review by Adam et al. in. 2023, reported that secondary PR performed four years after an initially retained patella produced significant improvements in Knee Society, Lonner and Feller scores, confirming that unresurfaced patellae can later compromise function [14]. Nardelli in 2023, reported on 1,209 nonresurfaced TKAs, a fivepercentagepoint drop in 10 year implant survival when severe preoperative patellofemoral OA (Iwano 3–4) was left untreated [18]. On the other hand, it’s reassuring to note that functional parity can be achieved in wellaligned cruciateretaining constructs with limited cartilage damage. In a bilateral series, Ko et al. in 2022 resurfaced only patellae graded Outerbridge ≥ 2 and found no PROM differences at 5.7 years [15]. A larger matched cohort of 500 CRTKAs by Noh et al. in 2022 also detected no significant differences in different functionality scores at three years. However, a positive patellarcompression test was more frequent when the patella was left intact (*p* = 0.01) [16]. Similar conclusions were reported by Samih et al. in 2023, 106 patients who underwent TKA and reported pain after TKA, in the evaluation of Knee Society, and Oxford scores as well as comparable early revision rates [12]. Sato et al. in 2021, performed a longitudinal study using a 3 T MRI on 38 patients who underwent conservative anterior cruciate ligament (ACL) arthroplasty without resurfacing. They measured the thickness of the patellar cartilage at discharge and at one, three, and five years. The authors observed that the mean thickness decreased from 2.8 mm to 1.3 mm (−54%) over five years, with the greatest loss occurring during the first 12 months. They also found that thinning correlated strongly with the occurrence of new anterior pain (*r* = 0.62). The authors concluded that leaving the patella intact may cause significant degeneration and recommended resurfacing or selecting patellae with borderline cartilage for PR to prevent medium-term pain and structural deterioration [13].

In a retrospective study performed in South Korea in 2022, 500 patients who underwent TKA were evaluated. Both techniques were compared (RP vs. WPR techniques), with similar clinical and functional results in the postoperative evaluation [16]. Ha et al. reported in 2019 a bilateral randomized trial, where they found comparable functionality scores but significantly less anterior knee pain. Other authors who performed a retrospective study in Germany in 2023, evaluating and comparing the functionality of both techniques and the return to sports activity; no differences were observed between the techniques used in performing TKA [20]. However, in 2019, Ha et al. reported that surgeons should consider routine or at least selective PR, especially in symptomatic patellae [21].

In 2022, Grela et al. reported that both resurfacing techniques can be effective in TKA, depending on the patient’s condition and preoperative factors. They recommended resurfacing as a viable option for patients with specific risk factors and anatomical characteristics [7]. Some authors advocate a selective resurfacing approach, which has been linked to reduced pain and lower rates of subsequent complications [5, 6]. Teel et al. [9] found that WPR techniques are linked to an increased risk of reoperation. No significant differences were noted in terms of anterior knee pain, satisfaction, or PROMs. 

Together, these findings indicate that patellar resurfacing provides small but durable functional gains, an apparent reduction in anterior knee pain, and fewer patellarelated reoperations. In contrast, secondary resurfacing can mitigate symptoms when initial retention fails, albeit with inferior long-term survival rates. The overall evidence therefore supports an individualised, pathologybased strategy. This strategy, which recommends resurfacing patellae with advanced cartilage loss or maltracking and retaining those with minimal degeneration when alignment is ideal, should instill confidence in our decision-making process. Scientific literature indicates that pain is a critical factor to monitor after surgery, as it can temporarily limit patients’ activities and, if persistent, prompt closer follow-up for possible revision surgery; some individuals may even refuse further procedures because of previous painful experiences and complications. In 2016, the Department of Orthopaedic Surgery at Haukeland University Hospital conducted a study of 308 knees in which they evaluated secondary resurfacing techniques. These patients were evaluated for persistent postoperative pain, and an improvement in function and pain was observed in patients who underwent PR compared to those who did not. In addition, improvement was observed after surgery, and superior clinical and functional outcomes were achieved. Therefore, performing a PR improves quality of life, functionality, and postoperative pain, although this is important for implant survival [8]. These results reinforce the idea that it is important to consider the possibility of revision if symptoms persist, mainly pain. However, the variability of results suggests that a selective strategy may be most appropriate, allowing treatment to be tailored to each patient’s specific characteristics. In our study, we observed an improvement in functional scores and postoperative pain scores. A recent study by Choi et al. in 2022 conducted a systematic review of six simultaneous bilateral RCTs, each involving 16 to 60 patients. In each study, one knee was assigned to receive PR, while the contralateral knee was designated as the WPR control. The findings indicate that the symptomatic benefits of PR may not be noticeable to patients when both knees are otherwise similar. This reinforces the argument for selective resurfacing rather than routine procedures [21]. The WPR technique may be a viable option for some patients; however, the potential risks of patellar wear and postoperative pain could impact the feasibility of secondary resurfacing. This analysis recommends that the decision to proceed with PR should be based on a personalized approach, taking into consideration relevant factors such as patellar cartilage quality, degree of wear, patellar maltracking, as well as the patient’s activity level and risk of potential complications, along with strategies to address them. It is also important to note that not all patients undergoing a non-resurfacing technique of PR experience anterior knee pain. However, it is essential to consider possible alterations in other knee components, particularly the situation of the extensor apparatus, patellar tracking, range of motion, and radiographic evaluation. In 2023, a study was conducted in Austria on 1209 patients who underwent the WPR technique, evaluating patients with advanced and moderate stages of patellofemoral OA; they reported patients who underwent PR with better survival rates and functional outcomes, as well as verification of proper patellar follow-up to avoid future complications. As we have observed, one of the most common complications related to the lack of PR is the development of anterior knee pain, which may lead to revision surgery and secondary resurfacing [18]. On the other hand, in 2023, Samih et al. performed a retrospective study including 106 patients who underwent TKA and compared both PR techniques. The most common complications in the PR group were as follows: infection (17.2%), persistence of anterior pain (13.7%), gait disturbance (6.8%), and loosening of the patellar component (3.4%). In the WPR group, fewer complications were observed. However, despite reducing the risks as mentioned earlier, anterior knee pain remains a significant concern [12]. While PR can alleviate anterior knee pain, it is not without inherent complications. In 2023, Adam et al. reported the most common complications associated with PR in TKA, including patellar fracture, patellar instability, and patellar maltracking. These complications may require revision surgery. Fracture of the patella is a serious complication in the medical field, impacting postoperative function and treatment difficulty, especially in patients who have undergone arthroplasty due to the recovery time and temporary functional limitation of TKA [14]. Research on complications related to PR indicates that the incidence of complications is similar to or lower than that associated with WPR technique. This research specifically identifies complications such as persistent pain, periprosthetic infection, and instability, with no studies suggesting that the PR technique in TKA carries an increased risk [4], [5], [6], [7], [14], [18]. In 2019, a study was conducted in China comparing PR and WPR techniques in patients with bilateral TKA. The study, which included 132 patients, observed complications associated with fractures and alterations of patellofemoral tracking in both groups. The study found that 23% of patients who did not receive resurfacing techniques experienced persistent anterior knee pain, compared to 19%. Both study groups encountered complications, though these were more prevalent in the WPR group. Despite the risks mentioned earlier, no complications such as patellar subluxation or dislocation, rupture of the quadricipital tendon, aseptic loosening, patellar osteonecrosis, or fractures were identified [19]. In Tokyo, Choi et al. reported a systematic review of bilateral randomized trials in 2022. This study reported similar results in patients with PR and WPR techniques and revision surgery. As previously mentioned, while the functional and pain benefits may be relevant, it is imperative to consider the risk of complications inherent to this technique [21]. The studies by Parsons et al. in 2021 and Fleaca et al. in 2022 reported a decreased frequency of revisions related to PR in TKA [6], [10]. Numerous scientific studies support PR as a technique that improves implant survival and reduces the incidence of anterior knee pain, a common cause of revision surgery. Sometimes PR in TKA is an optional technique, the preferred treatment for improving knee function and relieving pain in patients with an advanced degenerative stage. Our ARR and NNT values demonstrate the practical relevance of PR. They indicate that PR is sufficient to treat 14 knees to prevent loosening and just over one knee to avoid persistent anterior pain.

The patella plays a crucial role in TKA, and adequate treatment is essential to ensure survival, reduce complications, and minimize the need for revision surgery. In 2023, Shah et al. found no differences in the limited reviews within their meta-analysis on kneeling ability, highlighting the absence of reliable survival data associated with resurfacing, and no difference in patellarrelated revision [11]. In 2019, Allen et al. reported that patients WPR technique had fewer complications related to the patellar component, such as fractures and maltracking. This technical fact may reduce some patients’ need for medical revisions [17]. Conversely, the WPR technique has been promoted as a viable option in some instances, especially in patients with good-quality patellar cartilage or those with mild patellar OA. However, recent findings indicate that the WPR may be linked to a decreased implant survival rate in patients with more advanced patellofemoral OA, along with an elevated risk of persistent pain and the necessity for revisions [13]. In 2023, Nardelli et al. presented a study in Austria that compared both techniques in TKA. This study showed a 10-year survival rate of 93.3% in the PR group and 88.6% in the WPR group [18].

In 2001, Barrack et al. conducted a double-blind trial and confirmed that neither technique nor comorbidities predicted persistent anterior pain [22]. Similarly, Pehlivanoglu et al. (2019) compared patients with primary and RA and found no significant difference in anterior pain after patella preservation [23]. A Japanese study of patients with RA also showed improved quality of life and reduced pain when climbing or descending stairs after undergoing patellar resurfacing [24]. Benazzo et al. (2020) concluded that cases involving inflammatory arthritis, patellofemoral pain when ascending stairs, and female patients—all of which are associated with an increased risk of anterior pain—should be prioritized for PR [5]. Holt et al. (2006) examined 30 knees affected by RA that were treated without resurfacing. They found that the mean Visual Analog Scale (VAS) pain score decreased significantly from 7.3 to 1.5 after two years, indicating approximately 79% pain relief. Additionally, there was no need for resurfacing procedures afterward [25]. Benazzo et al. (2020) emphasize that to minimize complications, the decision to perform resurfacing should be based on factors such as patellar bone quality, angular deformity, and tracking follow-up. For patients with rheumatic conditions, they recommend resurfacing when other options—including patellar denervation, osteophyte resection, articular surface remodeling, or deep synovectomy of the quadriceps tendon are not viable [5]. Finally, according to Adam et al. in 2023, reported that patients with inflammatory rheumatic pathologies and those affecting synovial tissue or cartilage are suitable candidates for resurfacing techniques in TKA procedures [14].

Patients with rheumatologic pathologies require particular attention due to the chronic use of immunosuppressive drugs. These medications have the potential to impact the healing process, increase the risk of infection, and influence the recovery and outcomes of the procedure. A comprehensive investigation was conducted to identify the factors that contribute to complications. According to the 2022 recommendations of the American College of Rheumatology and the American Association of Hip and Knee Surgeons regarding the preoperative treatment of rheumatologic patients and arthroplasty procedures, the suspension and resumption of immunosuppressive therapies are recommended. To ensure a smooth transition from biologic agent suspension to resumption upon completion of the healing process, it is strongly recommended that methotrexate and hydroxychloroquine be administered during the preoperative period. In addition, medications that allow better control of specific pathologies are also advised. It is essential to taper glucocorticoids gradually to avoid triggering adrenal insufficiency and, consequently, to minimize the risk of infections [26]. However, a multidisciplinary approach is recommended; the goal is to reduce complications and improve long-term outcomes. 

To date, no study has definitively established PR as an independent prognostic factor in primary TKA, yet our data provide compelling evidence in that direction. The OR and RR for both aseptic loosening (OR 0.34; RR 0.37) and anterior knee pain (OR 0.004; RR 0.03) were markedly below 1, indicating that the absence of resurfacing carries a significantly higher risk of these complications. Both associations were statistically significant (*p* < 0.05). The confidence interval for anterior pain was narrow (0.01–0.08), reflecting a precise estimate, whereas the interval for loosening (0.15–0.92) was broader but still excluded unity. By contrast, the relative effect on all-cause revision was small and not significant (RR 0.77, ARR − 1.6%, *p* = 0.59), and the multivariable Cox model likewise found that the absence of PR was not an independent predictor of failure (HR 0.80; 95% CI 0.01–5.00; *p* = 0.76). Instead, aseptic loosening emerged as the dominant driver of revision (HR 16.7; *p* < 0.001).

The main limitation of this study is its setting in a tertiary, high-volume arthroplasty centre that treats a high proportion of complex rheumatologic cases (approximately 85% RA, 80% female). This case mix may limit generalisability to community hospitals serving predominantly osteoarthritic populations. The restricted demographic diversity, coupled with the single-centre design, could introduce selection bias. Nevertheless, the surgical technique—posterior stabilized femorotibial components with a cemented polyethylene patellar button—mirrors contemporary global practice, and our findings are consistent with those of large international registries, supporting their broader relevance. Although 80% of patients had concomitant hip osteoarthritis, only 3.3% reached grade III. Excluding them did not alter the results, suggesting that referred pain has a marginal effect on our estimates.

Across the 20-year cohort, PR achieved an absolute risk reduction of 83% for persistent anterior knee pain (NNT ≈ 2) and 7.5% for aseptic loosening (NNT ≈ 14), without increasing overall revision risk. These benefits, corroborated by sensitivity analyses, suggest that the principal mechanism is improved patellofemoral congruence rather than tibiofemoral protection. Taken together with recent meta-analyses showing similar reductions in pain and reoperation, our data support a selective resurfacing strategy—targeting patellae with advanced cartilage loss or maltracking—over a blanket policy.

Future prospective multicenter studies are required to confirm whether the magnitude of pain reduction and loosening prevention is retained in lower-risk, predominantly osteoarthritic cohorts, and to evaluate these effects in newer cementless implant designs.

## Conclusion

In this series of 271 primary TKAs, the PR technique provided an absolute reduction of 83% in anterior knee pain and 7.5% in aseptic loosening without increasing the overall revision rate. This benefit was confirmed by OR, RR and a multivariable Cox model, in which loosening, rather than absence of PR, emerged as the only independent predictor of reoperation.

To our knowledge, this is the first longitudinal investigation explicitly testing PR as an independent prognostic factor for global aseptic loosening, thereby filling a persistent gap in the arthroplasty literature. These findings support the routine consideration of patellar resurfacing when patient-, implant-, and era-related variables are taken into account, and offer a biologically plausible mechanism reduction of patellofemoral maltracking and shear forces to explain the observed survival benefit. These findings support the recommended practice of PR, at least for patients at risk of patellofemoral pain or with high functional demands, while recognizing that larger prospective studies are needed to define its impact on very long-term prosthetic survival. Based on these findings, the department adopted a selective resurfacing protocol in 2023: patellae with Outerbridge ≥ 2 or trackoff-center after trial reduction are resurfaced.

There are no precise indications for decision-making regarding resurfacing the patella during TKA. Our study found that PR during primary TKA offers patients the best functionality and pain relief results. This technique has been shown to reduce postoperative complications and improve patient’s quality of life. 

## Supplementary Information

Supplementary material 1: Table 1.

Supplementary Material 2: Table 2.

Supplementary Material 3: Table 3.

Supplementary Material 4: Table 4.

Supplementary Material 5: Table 5.

Supplementary Material 6: Table 6.

Supplementary Material 7: Table 7.

Supplementary Material 8: Table 8.

Supplementary Material 9: Figure 1.

Supplementary Material 10: Figure 2.

Supplementary Material 11: Strobe checklist.

## Data Availability

The datasets generated and/or analysed during the current study are available in the Zenodo repository. All data about this research is available on https://zenodo.org/records/15570904.

## Abbreviations

ACL: Anterior cruciate ligament
ARR: Absolute risk reduction
CR: Cruciate‑retaining
HR: Hazard ratio
IC: Confidence interval
IQR: Interquartile range
KSS: Knee Society score
ND: No statistical difference
NNT: Number needed to treat
NRS: Numeric rating scale
NS: significant
OA: Osteoarthritis
OKS: Oxford knee score
OR: Odds ratio
PR: Patellar resurfacing
PROMs: Patient-reported outcome measures
QALYs: Quality-adjusted life years
RA: Rheumatoid arthritis
RCTs: Randomized controlled trials
RR: Risk ratio
SLE: Systemic lupus erythematosus
THA: Total hip arthroplasty
TKA: Total knee arthroplasty
TKAs: Total knee arthroplasties
VAS: Visual Analog Scale
WOMAC: Western Ontario and McMaster Universities Osteoarthritis index
WPR: Without patellar resurfacing
Δ: The magnitude of the difference
