# Supplementary figures and images for: Patellar resurfacing as a prognostic variable in total knee arthroplasty: a two‑decade retrospective cohort (2000‑2020)
Source: BMC Musculoskelet Disord. 2026 Jan 13;27:27. doi: 10.1186/s12891-025-09076-y (PMC12801448; doi:10.1186/s12891-025-09076-y)

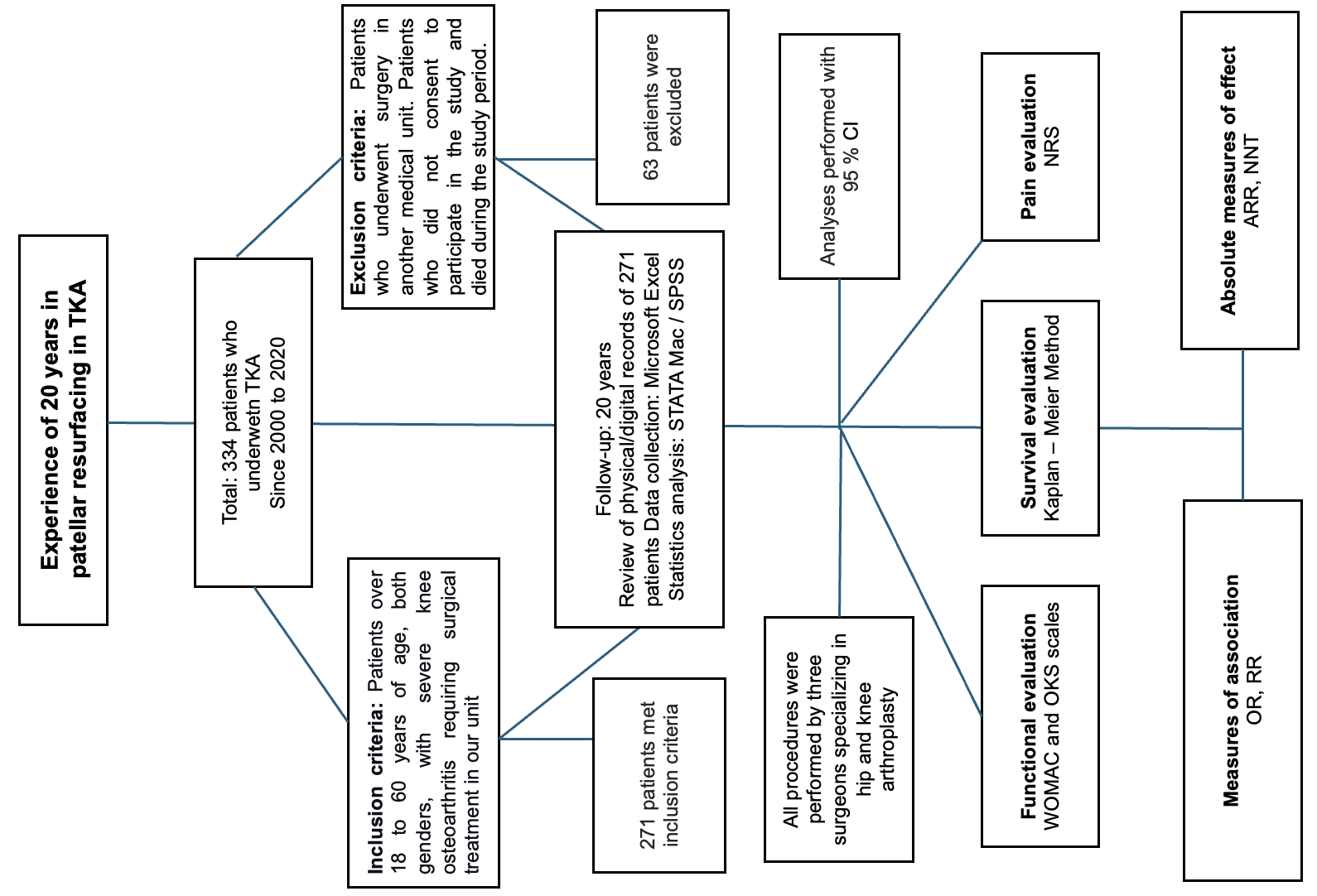

Supplement: Supplementary file 9 — Supplementary Material 9: Figure 1. [file 12891_2025_9076_MOESM9_ESM.png]
