# Supplementary material for: Patellar resurfacing as a prognostic variable in total knee arthroplasty: a two‑decade retrospective cohort (2000‑2020)
Source: BMC Musculoskelet Disord. 2026 Jan 13;27:27. doi: 10.1186/s12891-025-09076-y (PMC12801448; doi:10.1186/s12891-025-09076-y)
Supplement: Supplementary file 11 — Supplementary Material 11: Strobe checklist. [file 12891_2025_9076_MOESM11_ESM.docx]

# STROBE Checklist – Compliance Audit (Manuscript v2)

| Domain / Item | No. | STROBE Recommendation | Current status & manuscript location | Action required |
| --- | --- | --- | --- | --- |
| Title / Abstract | 1a | Indicate study design in title | ✔ Title includes “Two‑Decade Retrospective Cohort (2000–2020)” | – |
| Title / Abstract | 1b | Informative, balanced abstract | ✔ Lines 3‑40 give design, sample, main findings; still add CI for HR loosening | Add 95 % CI for HR loosening in Abstract |
| Introduction | 2 | Background / rationale | ✔ Lines 75‑160 provide context & controversy | – |
| Introduction | 3 | Specific objectives & hypotheses | ✔ Lines 161‑171 state objective + hypothesis | – |
| Methods | 4 | Present key elements of design early | ✔ Lines 175‑188 (“retrospective comparative cohort…”) | – |
| Methods | 5 | Setting & relevant dates | ✔ Lines 189‑203 (hospital, 2000‑2020, FU to 2024) | – |
| Methods | 6a | Eligibility, sources, follow‑up | ✔ Lines 204‑222 give criteria, follow‑up med 8 y | – |
| Methods | 6b | Matching (if any) | n/a | – |
| Methods | 7 | Clearly define variables, outcomes, confounders | ✔ Lines 223‑246 list exposures/outcomes; confounders age, BMI, RA | – |
| Methods | 8* | Data sources / measurement for each variable | ✔ Lines 247‑255 describe operative note, registry, radiographs | – |
| Methods | 9 | Address potential bias | ✔ New subsection “Bias control” lines 301‑315 | – |
| Methods | 10 | Study size rationale | ✔ New paragraph “Study size” lines 316‑325 (pragmatic cohort; post‑hoc power) | – |
| Methods | 11 | Handling of quantitative variables / groupings | ✔ Lines 256‑268 (age bands, BMI WHO, Follow‑up IQR) | – |
| Methods | 12a | Statistical methods incl. confounding control | ✔ Lines 269‑300 (Kaplan–Meier, Cox adj.) | – |
| Methods | 12b | Subgroup / interaction methods | ✔ Sensitivity hip‑OA model lines 210‑213 | – |
| Methods | 12c | Missing data | ✔ Sentence added “Missing‑data handling” lines 326‑329 | – |
| Methods | 12d | Loss to follow‑up | ✔ Deaths & exclusions lines 204‑212; censoring described | – |
| Methods | 12e | Sensitivity analyses | ✔ Hip‑OA sensitivity; but not labelled as such | Label as “Sensitivity analysis” in Methods |
| Results | 13a | Numbers at each stage | ✔ Flow diagram Fig 1 + lines 311‑318 | – |
| Results | 13b | Reasons for non‑participation | ✔ Lines 312‑318 list deaths/declined/elsewhere | – |
| Results | 13c | Flow diagram | ✔ Figure 1 present | – |
| Results | 14a | Participant characteristics & confounders | ✔ Table 1 + 2 | – |
| Results | 14b | Missing data per variable | Not tabulated |  |
| Results | 14c | Follow‑up time summary | ✔ Median 8 y (IQR 4–15) line 212 | – |
| Results | 15 | Outcome event numbers over time | ✔ Tables 3‑7, Kaplan–Meier | – |
| Results | 16a | Unadjusted & adjusted estimates with CI | ✔ Table 7 & Cox model Table 8 | – |
| Results | 16b | Category boundaries for categorised variables | ✔ Age/BMI categories defined lines 256‑263 | – |
| Results | 16c | Translate RR to absolute risk | ✔ ARR & NNT lines 380‑390 | – |
| Results | 17 | Other analyses (subgroups, sensitivity) | ✔ Hip‑OA; implant‑era Table 5; but label needed | Rename subsection “Subgroup & sensitivity analyses” |
| Discussion | 18 | Key results vs objectives | ✔ Lines 600‑620 opening Discussion | – |
| Discussion | 19 | Limitations | ✔ Lines 700‑720 (single‑centre, RA case‑mix) | Quantify bias direction where possible |
| Discussion | 20 | Interpretation considering evidence | ✔ Lines 720‑780 | – |
| Discussion | 21 | Generalisability | ✔ Lines 710‑725 mention external validity | – |
| Other information | 22 | Funding | ✔ Lines 820‑825: “No funding” | – |

Legend
✔ = item adequately addressed
All other STROBE elements are covered.
